# Established Yazd human foreskin fibroblast lines (#8, #17, and #18) displaying similar characteristics to mesenchymal stromal cells: A lab resources report

**DOI:** 10.18502/ijrm.v20i7.11554

**Published:** 2022-08-08

**Authors:** Fatemeh Hajizadeh-Tafti, Jalal Golzadeh, Ehsan Farashahi-Yazd, Hassan Heidarian-Meimandi, Behrouz Aflatoonian

**Affiliations:** ^1^Stem Cell Biology Research Center, Yazd Reproductive Sciences Institute, Shahid Sadoughi University of Medical Sciences, Yazd, Iran.; ^2^Abortion Research Center, Yazd Reproductive Sciences Institute, Shahid Sadoughi University of Medical Sciences, Yazd, Iran.; ^3^Department of Reproductive Biology, School of Medicine, Shahid Sadoughi University of Medical Sciences, Yazd, Iran.; ^4^Medical Nanotechnology and Tissue Engineering Research Center, Yazd Reproductive Sciences Institute, Shahid Sadoughi University of Medical Sciences, Yazd, Iran.; ^5^Department of Advanced Medical Sciences and Technologies in Medical Sciences, School of Paramedicine, Shahid Sadoughi University of Medical Sciences, Yazd, Iran.

**Keywords:** Cell therapy, Fibroblasts, Mesenchymal stem/stromal cells, Human embryonic stem cells, Micro-vesicles.

## Abstract

**Background:**

Fibroblasts from different parts of the human body have been used in cell biology, drug discovery and cell therapy studies. One of the most available sources of human fibroblasts is neonatal foreskin. Not only do these cells have wound-healing applications, but they are also the most popular source for pluripotent stem cell biotechnology. Moreover, several studies have indicated that different sources of fibroblasts display similar features to mesenchymal stem cells.

**Objective:**

Generation and establishment of new human foreskin fibroblast cell lines called Yazd human foreskin fibroblasts (YhFFs).

**Materials and Methods:**

In this lab resources study, the production of 3 YhFF cell lines (YhFF#8, YhFF#17, and YhFF#18) is reported. Their biological features were characterized using immunofluorescence, polymerase chain reaction, and flow-cytometry for mesenchymal markers such as fibronectin, vimentin, CD44, CD73, CD90, CD105, and hematopoietic markers CD34 and CD45.

**Results:**

The YhFF cell lines were passaged more than 40 times and their normal karyotype was checked using G-binding. Similarly to previous reports, the flow cytometry analysis revealed that the YhFF cell lines displayed mesenchymal stromal cell characteristics.

**Conclusion:**

This study will contribute to the development of clinical-grade cell-based products such as micro-vesicles and exosomes for future therapeutic applications in regenerative medicine.

## 1. Introduction

Fibroblasts are elongated, spindle-shaped cells with high proliferative and migration potential. They are the major cellular parts of connective tissues with multiple biological functions. They communicate with neighboring cells and tissues through secreting extracellular matrix molecules, growth factors, cytokines, and chemokines (1). One of the human fibroblasts is human foreskin fibroblasts (hFFs) that can be isolated from neonatal and adult foreskin tissues. These hFFs can be used as allogeneic candidates for wound healing, particularly in diabetic cases in which skin fibroblast generation is affected by the diabetes mellitus (2).

Following the first derivation of human embryonic stem cells (hESCs) in 1998 using mouse embryonic fibroblasts (3), hFFs have been used as a human source feeder layer (4, 5) to prevent the risk of animal pathogen transmission to hESCs and their derivatives for future cell-based therapeutic applications (6). Moreover, due to the production of the leukemia inhibitory factor by mouse embryonic fibroblasts, these cells can support the generation and expansion of mouse embryonic stem cells without adding exogenic leukemia inhibitory factor to the culture (7). Furthermore, hFFs have been introduced as a highly efficient source for generating induced pluripotent stem cells (PSCs) (8).

Interestingly, some reports have indicated that fibroblasts display similar characteristic markers to mesenchymal stem/stromal cells (MSCs) excluding the differentiation capacity and colony formation capability (1, 9). In addition, exosomal therapy has paved a new approach in regenerative medicine using MSC sources (10, 11). Given the important role of different sources of human fibroblasts, especially hFFs, herein we report our generation of Yazd hFFs named YhFF cell lines (#8, #17, and #18) which have been used already to support Yazd hESC line derivation and culture.

## 2. Materials and Methods

The chemicals were obtained from Sigma-Aldrich, Poole, UK and the culture medium and supplements from Invitrogen, UK, unless otherwise stated.

### Sample collection and patient information

In this lab resource study, 18 individual samples of human neonatal foreskin were collected from Madar hospital, Yazd, Iran. The fresh samples were placed in 2 ml of Dulbecco's Modified Eagle Medium (DMEM) containing 10% foetal bovine serum (FBS) and antibiotics, and coded to maintain anonymity, and then were transferred to the laboratory.

### Preparation of neonatal foreskin samples

The neonatal foreskin sample was washed in a Petri dish in DMEM/10% FBS/antibiotic and minced into small pieces. The neonatal foreskin pieces were then treated overnight in 0.1% collagenase type I in DMEM/10% FBS/antibiotic at 37 C in 5% CO ${}_{2}$ . Subsequently, cells were recovered by aspiration and washed by centrifugation for 3 min at 200 g. The supernatant was discarded and the pellet was recovered for the generation of YhFFs.

### Establishment and banking of YhFFs

Some of the samples were used to generate skin derived precursor cells and keratinocytes. Most of the neonatal foreskin derived cells were attached to the growing surface the day after the initial cell seeding. Half of the culture medium was exchanged every 2 days. Initially, cells were proliferated partially from cluster-like outgrowths and partially from single cell clones. By day 4 of the culture, cell confluency was around 85-90% ready for passaging. The YhFFs were passaged enzymatically using Trypsin-EDTA, initially 1:2 into T25 flasks. Then each T25 flask of YhFFs was passaged 1:1 into the T75 flask. The expansion of the YhFFs depended on the proliferation rate. The cells were then passaged from 1:2-1:6 ratios. For the banking of the cells, the slow freezing method using 10% dimethyl sulfoxide/90% FBS as a freezing solution was applied. Following disaggregation of the cells (at 85-90% confluency in T75) and centrifugation, 600 µl of freezing solution was added to the pellet and cells were transferred into the 1 ml cryovials. Cryovials containing the YhFFs were transferred into Mr Frosty (Thermo Fisher Scientific, UK) and kept in a -80ºC freezer. The day after, the cryovials were coded with the name of the cells and passage number with a date, and were placed in liquid nitrogen tanks for the storage and banking of the cells. The location of the vials was recorded in the freezing datasheets. The YhFFs were passaged more than 40 times, which indicated that the YhFF cell lines are possibly immortal with unlimited proliferation capacity.

### Cell proliferation assay

The cell proliferation assay was done by counting the cells before seeding and after harvesting following the confluency of the flasks before the next passage. The proliferation rate of the different YhFF cell lines at late passages (33-36) was calculated and compared.

### Karyotype analysis of YhFFs

To investigate the chromosomal content of the YhFFs, the karyotype of the cells in metaphase was determined using a standard G-banding procedure as explained elsewhere. Briefly, cells were cultured in flasks for 5-6 days. Following treatment with colchicine (10 μg/ml), harvested YhFFs were stained using a standard G-banding technique. G-bandings were analyzed under light microscopy (Axiophot, Ziess, Germany) using applied spectral imaging software (4).

### Immunofluorescent localization of cell markers

The identification of fibronectin and vimentin was carried out using immunofluorescent localization, as previously described (12-14). In summary, cells were washed twice for 5 min in phosphate buffered saline (PBS) containing 1% FBS, followed by incubation in 0.1% Triton X in PBS for 5 min and then were incubated overnight at 4ºC with primary antibody. The day after, cells were washed twice in PBS and incubated with appropriate secondary antibodies for 1 hr at 37ºC. The preparations were covered with mounting medium (Vectashield; Vector laboratories, USA) or PBS and examined by microscopy using phase contrast and appropriate UV excitation optics. The details of the primary and secondary antibodies are listed in table IA.

### Flow cytometry

Following the trypsin treatment of the YhFF cell lines at passage numbers 14 (YhFF#8) and 34 (all cell lines; YhFF#8, YhFF#17, and YhFF#18), the YhFFs were washed with PBS containing 0.5% FBS. Then, the cells were labelled with CD105, CD73, CD90 and CD44 (BD Bioscience, San Jose, CA, USA) as primary antibodies for 30 min at 4 C, followed by incubation with the appropriate fluorescent-conjugated secondary antibodies in the dark at 4 C for 60 min. The samples were analyzed on BD fluorescence-activated cell sorting Calibur (BD Bioscience, New Jersey, USA). Data analysis was performed using the FlowJo 7.6 software.

### RNA isolation, cDNA production, and reverse transcription polymerase chain reaction (RT-PCR)

Similar to research by Akyash and co-workers, the YhFFs from the different lines (#8, #17, and #18) at passages 14 and 24 were collected. Total RNA was extracted after suspending the resulting pellets in 500 μl of TRI reagent (Sigma, USA) according to the standard protocol provided by the manufacturer. Total RNA was treated with DNase I (Thermofisher, USA) to remove genomic DNA. First-strand cDNA was synthesized using cDNA synthesis kit (Thermofisher, USA) and PCR was performed using prepared cDNA and primers of different genes (Table IB) by Taq 2x Master Mix Red 1.5 mm MgCl2 (AMPLIQON, Denmark). For PCR, samples were annealed for 5 min at 95 C for initial denaturation, followed by 40 cycles of 30 sec at 95 C, 30 sec at 58-60 C (Table IB), and 30 sec at 72 C. Finally, the PCR products were identified in 2% agarose gel electrophoresis (4).

**Table 1:** List of the antibodies and primers


**A**	**Primary antibody**				**Secondary antibody**				
	**Fibronectin**	1:200	Abcam ab6328		Anti-mouse IgG (FITC)	<1:100		Abcam ab6785	
	**Vimentin**	1:100	Millipore AB5733		Anti-mouse IgG (FITC)	<1:100		Abcam ab6785	
**B**	**Primers**								
	**Gene**	**Forward primer**		**Reverse primer**			<**Annealing temperature ( ${}^{\circ }$ C)**		**Product size (bp)**
	*bFGF*	CCAGAAAACCCGAGCGAG		GGCGTCACATCTTCTACATCTC			58		104
	*FN1*	AGGAAGCCGAGGTTTTAACTG		AGGACGCTCATAAGTGTCACC			58		106
	*VIM*	TCTATCTTGCGCTCCTGAAAAACT		AAACTTTCCCTCCCTGAACCTGAG			58		270
	* β 2M*	AGATGAGTATGCCTGCCGTG		TGCGGCATCTTCAAACCTC			60		106
*FN1*: Fibronectin, *VIM*: Vimentin, *bFGF*: Basic fibroblast growth factor, * β 2M*: Beta-2 microglobulin									

### Ethical considerations

The study protocol has been approved by the Shahid Sadoughi University of Medical Sciences Ethics Committee, Yazd, Iran (Code: IR.SSU.REC.1394.103). All participants were asked to complete the informed consent form.

## 3. Results

### Generation of YhFF cell lines 

**Figure 1:**
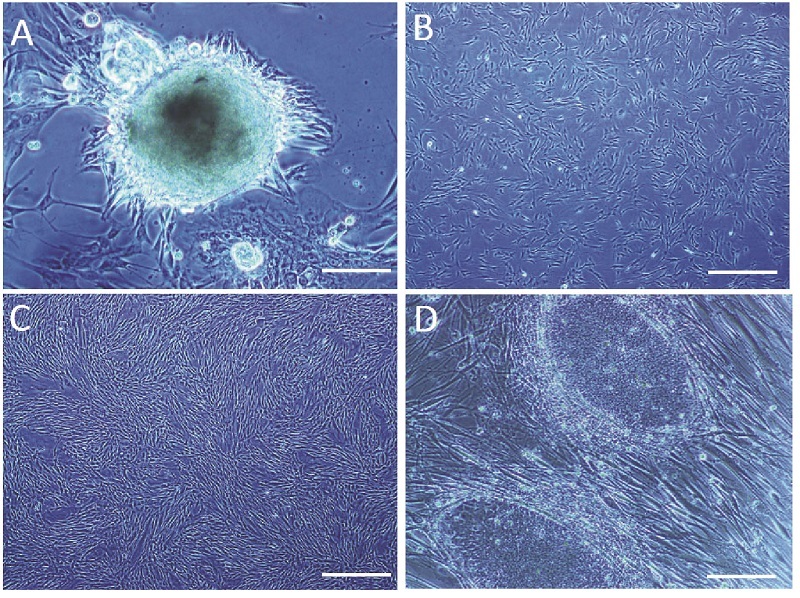
Establishment and propagation of YhFFs: Outgrowths of YhFFs from human neonatal foreskin samples following enzymatic treatments. Initially, some cells were proliferated from the cluster-like structure (A) and some were single cells (B). Following 3-5 days, flasks become confluent and ready for passage (C). Mitotically inactivated YhFFs were used as human feeder layer (D) for culturing Yazd hESCs (4). Scale bars: A = 50 µm, B and C = 200 µm, D = 100 µm.

18 neonatal foreskin samples were used to derive different cell types (keratinocytes, skin derived-precursor cells and fibroblasts). From 3 different neonatal foreskin samples, 3 fibroblast cell lines (YhFF#8, YhFF#17 and YhFF#18) were established. Initially, within the cultures, some clusters (Figure 1A) similar to those which are claimed to be putative pluripotent male germ line stem cells (9) were observed which later, following several passaging, formed more mesenchymal stromal phenotype (Figure 1B and 1C).

### YhFFs as a feeder layer to support Yazd hESC lines

YhFFs were used as a feeder layer for the derivation and expansion of the Yazd hESC lines (4). Initially the Yazd hESC lines were derived from mouse embryonic fibroblasts and then later, they (Yazd1-3) were expanded on the YhFFs (Figure 1D). Moreover, YhFF#8 was used for the derivation of new hESC lines (Yazd4-7) in xeno-free condition. From our experience, Yazd hESCs were less stressed on YhFF#8 as a feeder layer in culture. Both microdrop culture and open culture systems in well and flask scales were applied to the culture of the Yazd hESCs on YhFFs (4).

**Figure 2:**
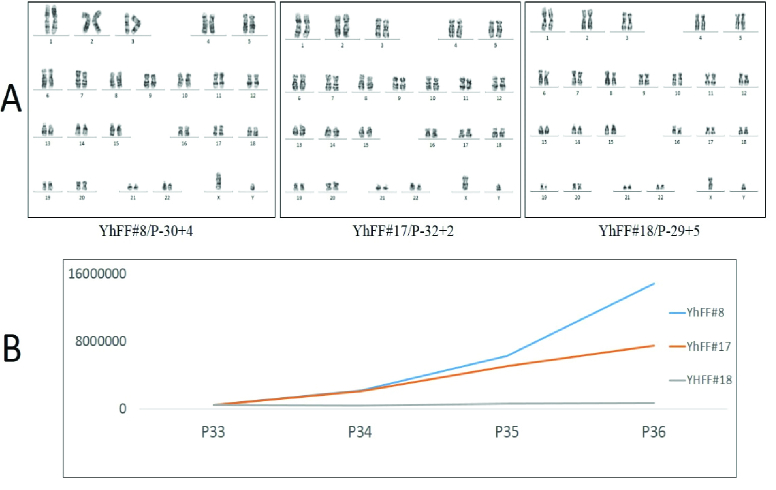
G-banding analysis for YhFF cell lines indicated the normal karyotype (46, XY) for all the cell lines at passage 34 (A). The multiplication rate of YhFFs at late passages (P33-P36) indicated that YhFF#8 cells had a good proliferation rate even in their late passages without signs of senescence (B).

The genetic content stability of the YhFF cell lines was examined using standard chromosomal G-banding staining, which showed normal male karyotype of the cells (Figure 2A). The population duplication rate (Figure 2B) of the cell lines (YhFF#17 and YhFF#18) was examined during 5 days of culture in which there was not a significant difference in the proliferation rate of the cell lines.

###  Mesenchymal stromal characteristics of the YhFFs

**Figure 3:**
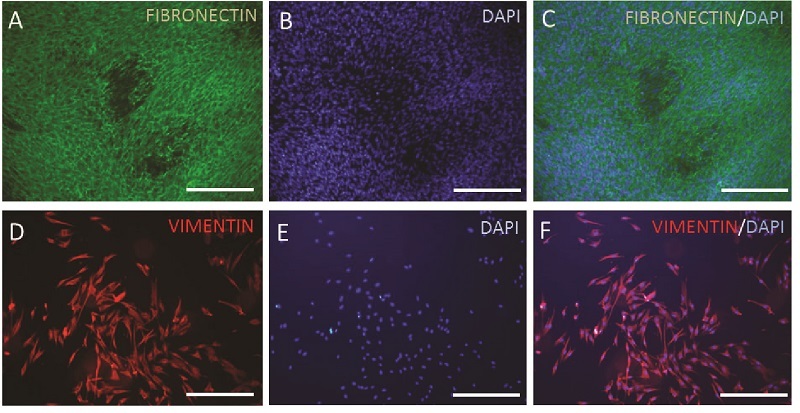
Immunofluorescence staining: Following cell proliferation, immunofluorescence staining indicated that *FN1* and *VIM* were expressed by the YhFFs #8 P14. Scale bars = 200 µm.

**Figure 4:**
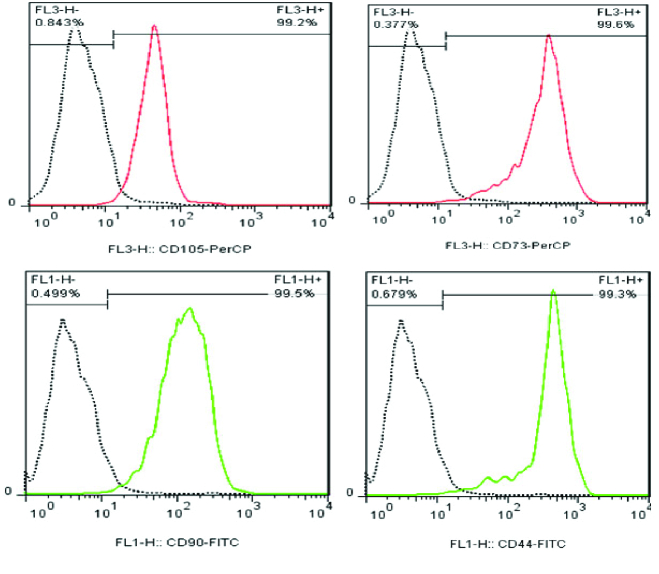
Flow cytometry analysis of YhFF#8 showed that more than 99% of the cells expressed CD44, CD105, and CD73 at passage 14.

The cultures of proliferating YhFFs showed expression of fibronectin (Figure 3A-C) and vimentin (Figure 3D-F). The flow cytometry data from passage 14 of YhFF#8 indicated similar characteristics of these cells to the MSCs given that the cells showed a high expression ratio of CD105, CD73, CD90, and CD44 markers (Figure 4).

**Figure 5:**
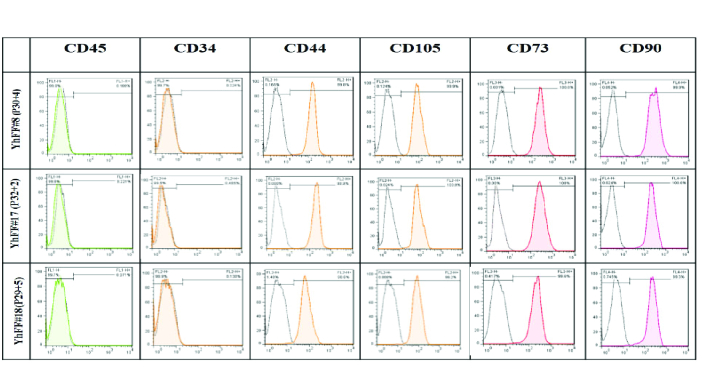
Flow cytometry analysis of the YhFF cell lines at passage number 34 showed cell characteristics similar to MSCs given the expression of CD44, CD105, CD73 and CD90 by the majority of the cells in the culture at late passage. The YhFFs did not express the hematopoietic markers CD34 and CD45.

The flow cytometry analysis revealed that the YhFF cell lines displayed similar characteristics as MSCs at passage number 34 (Figure 5).

**Figure 6:**
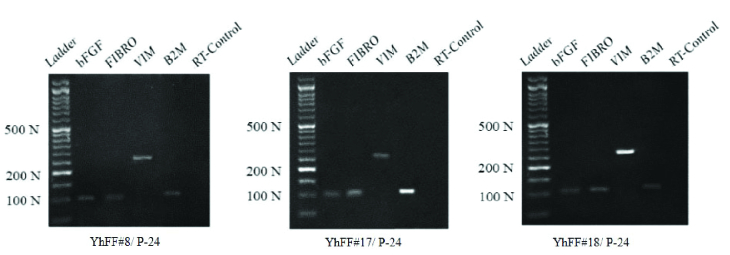
Reverse transcription polymerase chain reaction data: Gene expression profile of the YhFFs after passage 24 showed that all the cell lines expressed *bFGF* with *FN1* and *VIM*.

The RT-PCR gene expression profile of the different YhFF cell lines at passage 24 was assessed. To check the supportive role of the YhFFs in their high passage number (P24) for culturing hESCs, the expression of *bFGF *with* VIM *and* FN1* was checked and detected using RT-PCR (Figure 6).

## 4. Discussion 

Human fibroblasts will play critical roles in future cell therapy applications using PSCs. To use hESCs and their derivatives in clinics, human fibroblasts and especially hFFs are the best candidates as the human source feeder layer to keep the hESCs undifferentiated (5). Furthermore, for the generation of human induced pluripotent stem cells (hiPSCs) as the other counterpart of PSCs, human fibroblasts are the best available source for reprogramming (8).

In this work, 3 new hFF cell lines called Yazd hFFs (YhFF#8, YhFF#17, and YhFF#18) were produced. These cells have been used as a human source feeder layer to support Yazd hESC lines (Yazd1-3) in culture (4). Moreover, YhFFs were used to generate new Yazd hESCs (Yazd4-7) in xeno-free culture conditions. YhFFs are used for the generation of hiPSCs and so far, initial outgrowths of Yazd hiPSCs have been generated. Further expansions and characterization assessments of hiPSCs are in progress. YhFFs have been passaged more than 30 times while showing signs of unlimited proliferation capacity and immortalization. Interestingly, YhFFs have supported different passage numbers to keep the Yazd hESC lines proliferating while undifferentiated.

In the current study, the gene and marker expression of YhFFs were examined using different techniques. Fibronectin and vimentin, which are involved in cell motility and mobility (12, 15) as well as being characteristics of mesenchymal cells, were detected in YhFFs using immunofluorescence and RT-PCR. Several reports have shown the similarities between human fibroblasts and MSCs especially in their gene and marker expression profiles (1, 9, 16). The flow cytometry data confirmed that the YhFFs also expressed mesenchymal markers such as CD44, CD73, CD90, and CD105 in P14 and CD44, CD105, CD73, and CD90 markers in P34. YhFFs have already been used in our previous studies as a control group to test the mesenchymal properties of other cell types (17). Moreover, YhFFs have also been used as a feeder layer for the derivation and culture of Yazd hESCs (4). Additionally, YhFFs have shown that they have the capacity for reprogramming.

YhFFs were generated successfully and displayed similar characteristics to MSCs in gene and marker expression profile. Moreover, these cells were shown to be good candidates for PSC biotechnology. Further studies are being carried out using YhFFs to investigate their potential in future wound healing and exosome therapy applications.

## 5. Conclusion

3 hFF cell lines (YhFF#8, YhFF#17, and YhFF#18) were produced in this lab resources project which can be used in different in vitro and in vivo studies. Their derivatives such as conditioned medium, exosomes, and extracellular vesicles have regenerative medicine applications. Ultimately, this study will contribute to the derivation of clinical-grade cell-based products such as micro-vesicles and exosomes for future therapeutic applications in regenerative medicine.

##  Conflict of Interest

The authors declare that there is no conflict of interest.
